# Developing a Mindfulness Program for Families in a Pediatric Weight Management Clinic

**DOI:** 10.3390/children13050601

**Published:** 2026-04-27

**Authors:** Megan Lane, Bobby Verdugo, Natacha D. Emerson, Miranda Kim, Qiang Zhang, Christine K. Thang, Cambria L. Garell, Allison Gabriella Depas, Wendelin M. Slusser, Shanika Boyce, Alma D. Guerrero

**Affiliations:** 1 Department of Developmental Behavioral Pediatrics, University of California Los Angeles, Los Angeles, CA 90095, USA; 2 Division of Developmental Pediatrics, University of Tennessee Health Science Center, Memphis, TN 38163, USA; 3 Department of Psychiatry & Biobehavioral Sciences, University of California Los Angeles, Los Angeles, CA 90095, USA; 4 Semel Healthy Campus Initiative Center, University of California Los Angeles, Los Angeles, CA 90095, USA; 5 David Geffen School of Medicine, University of California Los Angeles, Los Angeles, CA 90095, USA; 6 Department of Pediatrics, University of California Los Angeles, Los Angeles, CA 90095, USA; 7 Department of Pediatrics, Charles R. Drew University of Medicine and Science, Los Angeles, CA 90059, USA; shanikaboyce@cdrewu.edu

**Keywords:** mindfulness, pediatric obesity, multidisciplinary clinic, program evaluation

## Abstract

**Background/Objectives:** Pediatric obesity is a public health epidemic in the United States and in many countries worldwide. Due to the interrelatedness of obesity and toxic stress, mindfulness is a promising practice to support healthful eating behaviors and combat stress in the management of this condition. In this pilot study we evaluated the acceptability and feasibility of implementing a brief mindfulness-based program for pediatric patients seeking treatment for overweight and obesity, with an assessment of exploratory outcomes. **Methods:** Nineteen children (ages 8–17 with body mass indices (BMIs) ≥ the 85th percentile) and caregiver dyads (*n* = 19) were recruited from a multidisciplinary pediatric weight management clinic. Four thirty-minute psychoeducational mindfulness-based sessions were provided via telehealth. Qualitative feedback was sought during and after program completion. Child and caregiver mindful eating and general mindfulness measures were collected from participant dyads at baseline, and one month and three months after program completion. **Results:** Qualitative program feedback from participants was generally positive. Session attendance rates were high (89%), with most participants highly engaged during sessions and the follow-up clinic visits. An analysis of exploratory measures data revealed no significant changes in child or caregiver dyad mindful eating or general mindfulness scores at one month (*n* = 9) or three months (*n* = 7) post-program completion compared to baseline (*n* = 10). **Conclusions:** This pilot, mindfulness-based program was feasible and acceptable to implement as a scalable behavioral intervention in long-term pediatric obesity treatment. Study of a larger, controlled sample is needed to determine the impact of program participation on mindful eating and general mindfulness, along with clinical obesity-related outcomes, in the management of pediatric obesity.

## 1. Introduction

Nearly one in five American children between the ages of 2 and 19 years old is categorized as obese based on their body mass index (BMI) [1], and one in three as overweight [2]. Affecting upwards of 15 million US children, pediatric obesity continues to be a public health challenge [3], often described as an *epidemic* due to its association with serious medical comorbidities and premature mortality [4,5,6,7]. Besides its association with conditions such as type 2 diabetes mellitus, hypertension, nonalcoholic fatty liver disease, obstructive sleep apnea and dyslipidemia, childhood obesity is also linked to psychological comorbidities including depression, anxiety, poor self-esteem and body image, and disordered eating [8,9,10]. Multidisciplinary services are recommended for children and adolescents with overweight and obesity [11].

Environmental risk factors for childhood obesity are numerous and far-ranging [2,12,13,14,15]. One important risk factor is *toxic stress*, a term that denotes the deleterious impact on physiology that results from long-term sympathetic system, or “fight or flight”, response [16]. Toxic stress has been shown to increase the risk for obesity via physiological, biochemical, cognitive, and behavioral pathways [17]. At the physiological level, toxic stress impacts the production of glucocorticoids, cytokines, and catecholamines, which in turn impact metabolic processes and an individual’s anthropometrics, such as BMI and waist circumference [18], and modulate the biochemical hormones ghrelin and leptin, which influence food consumption and satiety [19,20]. Adverse childhood experiences (ACEs), which are specific traumatic experiences in childhood, are established as robust risk factors for poor psychological and physical health. Children with a higher number of ACEs are more likely to develop toxic stress physiology and obesity [21].

Additionally, the psychological impact of stress predisposes individuals to obesity, as the cognitive load of chronic stress disrupts cognition and executive functioning [22,23], which negatively impacts health behaviors, including eating habits. While toxic stress is shown to predispose to obesity, obesity itself puts children and adults at risk for further experience of toxic stress, due to the high risk of experiencing weight stigma, bias, and discrimination [24,25,26]. Toxic stress and obesity often co-exist in a cyclical manner, making it important to address the causes and management of stress in obesity treatment.

Providing families with tools to combat toxic stress and to cope adaptively is paramount to breaking the vicious cycle of toxic stress and obesity. One health-promoting practice gaining attention across multiple domains is *mindfulness*. Mindfulness is a quality of consciousness characterized by continually attending to one’s moment-by-moment experiences, thoughts, and emotions with an open, non-judgmental approach [27]. Mindfulness-based interventions (MBIs) have been found to reduce stress and anxiety levels and improve vital signs such as heart rate and blood pressure [28,29].

In recent years, MBIs have attracted attention in modulating eating behaviors in adults and children, as they can help individuals become more aware of their thoughts and emotions around food, and of their eating and behavioral patterns [30,31]. Studies have found that MBIs can mitigate negative eating behaviors, including binge eating and emotional eating, and can promote weight loss [32,33]. Currently, the majority of studied MBIs as part of obesity management are in adult populations [34], creating the need for the development and study of MBIs targeting pediatric populations.

MBIs developed to prevent and address pediatric obesity show promise in improving eating attitudes and practices, and BMI outcomes [34,35]. However, most MBIs used in the treatment of pediatric obesity to-date have been delivered in a group-based format with lengthy curricula, including anywhere between 60 and 120 min long sessions over 6 to 16 weeks [34]. The time requirement of such programs limits accessibility for busy families experiencing competing social, financial, and educational priorities [36]. Lastly, most pediatric obesity MBIs in clinical settings have been facilitated by professional psychologists and psychology students, who are often limited in pediatric medical care settings [37,38,39,40].

To address the limitations listed above, we developed a four-module, individually delivered, family-based educational intervention, informed by evidence-based mindfulness practices related to family systems and eating behaviors, and delivered by a licensed clinical social worker. This pilot study describes the development, feasibility, and acceptability of a brief MBI called the Fit Mind program, which focuses on education about mindful behaviors and on empowering youth who are followed in a multidisciplinary weight management clinic. The Fit Mind program employs a mindfulness training conceptual model created by O’Reilly and Black [41], which focuses on obesity-related eating behaviors in children and adolescents. The Fit Mind program has four key themes, each weaving in principles to combat chronic stress and help participants make more mindful decisions when it comes to eating: (1) *Managing Stress*, (2) *Mindful Eating*, (3) *Mindful Thinking*, and (4) *Managing Cravings*. To ensure generalizability across clinical settings, the Fit Mind program was created to be deliverable by a wide range of pediatrics professionals, including licensed clinicians, allied health members, and trainees.

The primary objective of this pilot study was to assess the feasibility and acceptability of implementing an individually delivered MBI in the clinical practice of a busy, multidisciplinary pediatric weight management clinic. The secondary objective focused on exploratory outcomes, evaluating if there were changes in the participating groups’ mindful eating attitudes and practices and general mindfulness after completing the four-module Fit Mind course. It was hypothesized that the Fit Mind curriculum would be accessible to families, feasible to implement, and acceptable to partake in, and that scores on validated mindful eating attitudes and practices and general mindfulness rating scales among youth and caregivers would show an improvement after completion of the Fit Mind curriculum.

## 2. Materials and Methods

The Fit Mind MBI program was delivered in a multidisciplinary pediatric obesity clinic, at a large, urban, academic medical center’s Fit for Healthy Weight Clinic, e.g., the “Fit Clinic”. From prior analysis of the Fit Clinic’s patient demographics, about 50% of patients receiving care at the Fit Clinic are female and are of an average age of 13 years old [42]. The Fit Clinic’s patients initially have average BMIs of 34 kg/m^2^, with a 98% mean BMI percentile for age and sex, and 97% of patients’ BMIs fall in the obese range [42]. Approximately 13% of Fit Clinic patients report experiencing at least one ACE, based on prior retrospective chart review of the items listed in the 10-item Pediatric ACEs and Related Life Events Screener (PEARLS) questionnaire. The racial/ethnic data of the Fit Clinic patient population has not been analyzed due to frequent missing data and potential biases in collecting and documenting this information in the electronic medical record.

The Fit Clinic supports and treats children with childhood overweight and obesity, clinically defined as having a BMI ≥ 85th percentile and ≥95th percentile, respectively, for the child’s age and sex [11]. During a typical visit to the Fit Clinic, patients meet in-person with Fit Clinic team members, including a pediatrician, a child psychologist, and a registered dietitian. In-person, full-team visits typically occur every three months. In between full-team visits, patients meet with the clinic’s dietitian and/or psychologist on a bi-weekly-to-monthly basis, depending on individual needs. Connection with the Fit Clinic’s licensed clinical social worker is also available to address any specific psychosocial challenges. At each full-team visit, the clinic team members jointly collaborate to address the medical, psychosocial, and dietary factors impacting each patient and family.

### 2.1. Participants

Eligible study participants included both new and follow-up Fit Clinic patients, with a BMI ≥ 85th percentile for the child’s age and sex, along with their caregiver. Prospective participants of the study were approached and informed about the free, four-session Fit Mind program by Fit Clinic team members, during both new and follow-up Fit Clinic visits. Eligible participants were given the option to decline study participation and still participate in the four mindfulness-based sessions outside of this pilot study. To optimize engagement in the Fit Mind program, pediatric participants were given the option of participating alone or with their caregiver. Written informed consent was obtained from all legal guardians, and written assent was obtained from all pediatric participants in this study. To protect participant privacy, analysis of the pilot participants’ demographic data was not completed as part of this study. This study protocol was approved by the University of California Los Angeles (UCLA) Institutional Review Board (IRB #22-001689).

Inclusion criteria for pediatric participants included being between the ages of 8 and 17 years and families having reliable access to the internet to connect to virtual Fit Mind appointments. Patients who primarily speak English or Spanish were invited to participate. The minimum age of 8 years was chosen to ensure developmentally appropriate understanding of session content. Pediatric patients with acute psychiatric needs were referred to appropriate supports and excluded from this study; they were invited to consider participation in Fit Mind outside of the pilot study after establishing appropriate mental health care.

### 2.2. Study Procedure

**Figure 1:**
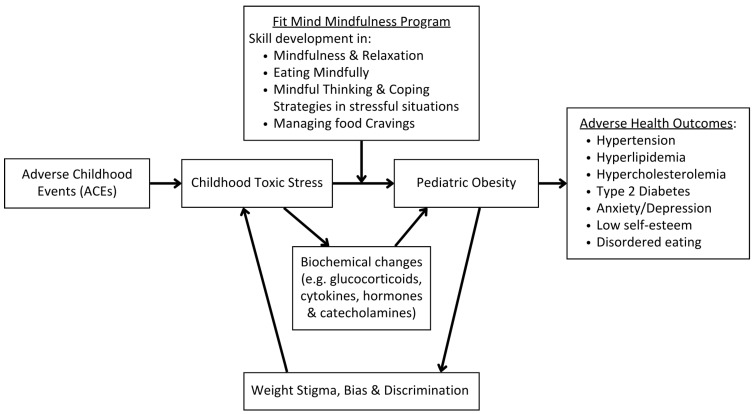
Conceptual model of Fit Mind’s impact on pediatric obesity treatment.

The Fit Mind MBI consisted of four consecutive, 30 min weekly telehealth sessions, totaling two hours of direct program delivery. Figure 1 illustrates the conceptual model of the Fit Mind program in addressing the cognitive and behavioral elements of obesity management with consideration of the interconnection of ACEs, toxic stress, and obesity. Fit Mind sessions were delivered to participants on an individual basis, either primarily to pediatric participants themselves, with caregivers checking in briefly at the beginning or wrap-up of each session, or to caregiver–child dyads together. The Fit Mind sessions were facilitated by the Fit Clinic’s licensed clinical social worker via a HIPAA-compliant institutional Zoom meeting, or via the Epic electronical medical records’ telehealth appointment system. The Fit Clinic’s licensed clinical social worker had extensive prior experience providing evidence-based mental health interventions to pediatric and adult populations in primary care settings. The Fit Mind sessions were conducted during the multidisciplinary Fit Clinic visits and billed for indirectly using multidisciplinary clinic codes.

Fit Mind was first delivered to five patients and their families between July and October 2022. These pediatric patients and their caregivers provided initial feedback regarding the feasibility, acceptability, and perceived usefulness of the Fit Mind program after their fourth and final Fit Mind session, through open-ended questions asked verbally by the Fit Mind program facilitator at the end of the final session. This feedback was incorporated into the development of the final Fit Mind curriculum used in the study described below. Subsequently, the Fit Mind pilot study was conducted between January and July 2023.

Participants were informed that the Fit Mind sessions were interactive in nature and contained questions, exercises, and activities for them to complete each week. Participants were also expected to set one health goal for the four-week period. Examples of health goals included becoming more aware of portion sizes, improving sleep hygiene, incorporating more fruits and vegetables into their daily intake, increasing physical activity, and practicing mindfulness during meals. Each goal was patient-initiated, with guidance from the facilitator, who assessed for potential barriers that might impact success.

**Figure 2:**
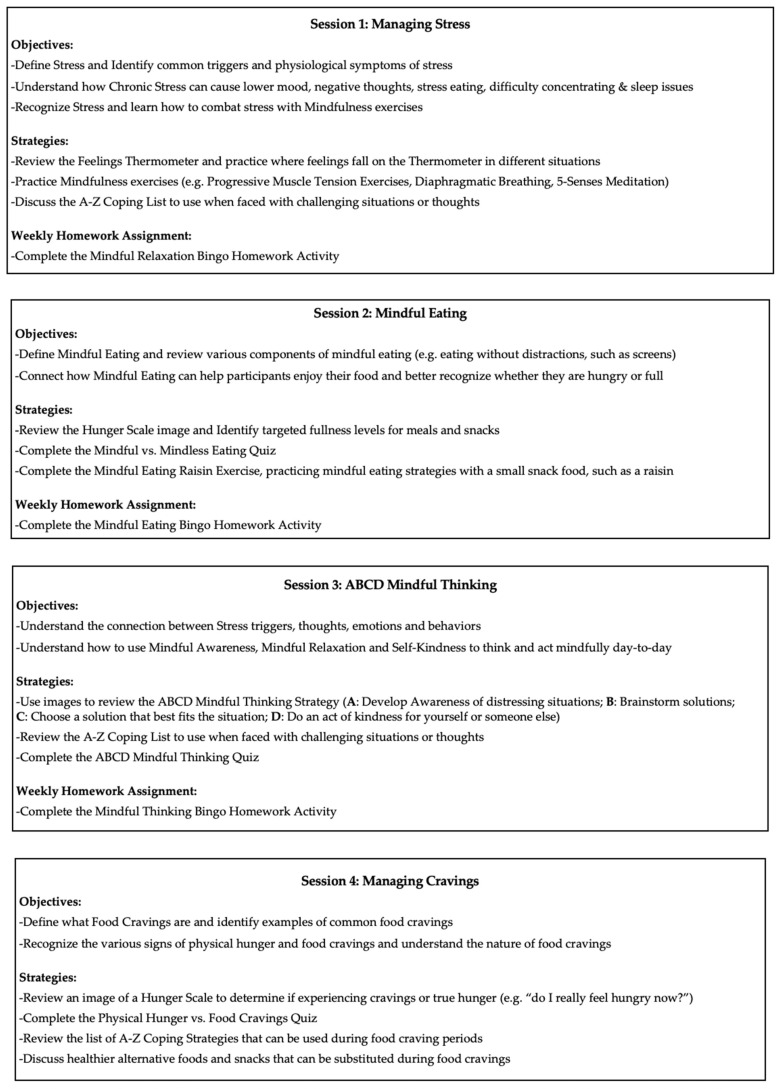
Topics, objectives, strategies, and homework assignments of the four Fit Mind sessions.

Four intervention components crucial in supporting pediatric populations with concomitant child obesity and ACEs were incorporated as the key themes of each of the program’s four modules, 1. *Managing Stress*, 2. *Mindful Eating*, 3. *Mindful Thinking*, and 4. *Managing Cravings*, as outlined in Figure 2. Evidence-based psychotherapeutic strategies including mindfulness, cognitive- and dialectical-behavioral approaches, problem-solving, and motivational interviewing techniques were woven into program delivery. These strategies were used to inform patient-centered goal creation, as homework to complete individually or with family in-between sessions. Homework in weeks 1–3 also involved completing family bingo activities relevant to the week’s topic (e.g., after Week 2, completing a bingo sheet that involved activities related to Mindful Eating). Each subsequent session started with a review of the previous week’s goal(s) and homework assignments. The unique objectives and strategies used in each session are detailed in Figure 2.

To present each week’s material, the Fit Mind curriculum was delivered visually and verbally, with images relevant to each week’s topics and activities presented through video-based screen sharing. The Fit Mind program facilitator delivered the modules in a standardized manner, completing the pre-determined activities within each of the four Fit Mind sessions (Figure 2). The Fit Mind facilitator manual is available from the authors on request. The program was delivered in either English or Spanish by the Fit Mind program facilitator, who is fluent in both languages, based on participant preference.

Attendance rates at Fit Mind sessions were collected. Pediatric and caregiver participants in the study were asked to complete validated mindfulness and mindful eating questionnaires at baseline and at 1 month and 3 months after completion of Fit Mind. Participants’ feedback of the Fit Mind program was verbally and informally solicited at the conclusion of their final Fit Mind session and/or at their first, full-team Fit Clinic visit after completion of the program. Informal observations regarding participant engagement during Fit Mind sessions and during participants’ subsequent, full-team Fit Clinic visits were made by the Fit Mind program facilitator and Fit Clinic team members, respectively, throughout the pilot study.

### 2.3. Measures

The following four questionnaires were completed anonymously by participants/caregivers at three time-points: (a) baseline (within 1 week of starting the Fit Mind program), (b) one month after completing the fourth and final Fit Mind session, and (c) three months after completing the Fit Mind program. Key themes that were examined through these surveys included attitudes and practices related to awareness of the physical and emotional sensations in relation to eating and food, as well as general mindfulness, related to the degree in which one behaves with awareness and accepts internal experiences without judgment. Questionnaires were administered through Qualtrics password-protected and HIPAA-compliant data entry web-based surveys. Questionnaires were completed by pediatric patients and their caregivers.

#### 2.3.1. Pediatric Questionnaires

Child participants completed two measures: the Mindful Eating Questionnaire-Child (MEQ-C) [43] and the Child and Adolescent Mindfulness Measure (CAMM) [44]. The MEQ-C is a 12-item questionnaire, with acceptable reliability and validity in youth, and includes two subscales (Mindless Eating and Awareness). The MEQ-C includes question items such as, “I notice the flavors in my food.” Each MEQ-C item is scored on a 4-point Likert scale from 1 (never/rarely) to 4 (usually/always). Each MEQ-C subscale score is calculated as the mean of items, and the summary score was the mean of the subscales. Higher scores on the MEQ-C signify a greater frequency of mindful eating behaviors.

The 10-item CAMM has been tested internationally and measures general mindfulness and acceptance among children and adolescents [45]. The items on the CAMM are rated on a Likert scale, ranging from 0 (never true) to 4 (always true). All items on the CAMM are reverse-coded so that a higher CAMM score reflects a higher level of general mindfulness.

#### 2.3.2. Caregiver Questionnaires

Caregivers completed two measures: the Mindful Eating Questionnaire (MEQ) [46] and the Cognitive and Affective Mindfulness Scale-Revised (CAMS-R) [47]. The MEQ is a 28-item adult counterpart to the MEQ-C pediatric mindful eating measure with good validity and reliability, and is commonly used as a primary mindful eating measurement in practice [48]. The MEQ includes five mindful subscales: Awareness, Disinhibition, Distraction, Emotional Response, and External Cues. MEQ responses are rated on a four-point Likert scale from 1 (never) to 4 (usually/always) to a series of statements related to eating behaviors such as, “I notice when there are subtle flavors in the foods I eat.” Each MEQ subscale score is calculated as the mean of items, and the summary score is the mean of the five subscales. Higher scores on the MEQ signify more mindful eating.

The CAMS-R is a 12-item questionnaire with acceptable psychometrics, measuring the degree to which individuals experience general mindfulness behaviors, such as present-focused attention, awareness, and non-judgmental acceptance of thoughts and emotions. CAMS-R items are rated using a 4-point Likert scale from 1 (rarely/not at all) to 4 (almost always). Higher CAMS-R scores reflect greater general mindfulness.

### 2.4. Data Analysis

Statistical analysis was completed using STATA version 17.0 software (StataCorp LP, College Station, TX, USA). Data from the online surveys reflect the child and parent scores on the MEQ, MEQ-C, CAMS-R and CAMM at baseline, prior to the Fit Mind program, at 1 month and 3 months post-intervention. We set out to analyze differences in scores between baseline and follow-up survey assessments. Mean rating scores between baseline and 1 month post-intervention and baseline and 3 months post-intervention were compared using two-sample *t*-tests assuming unequal variances, with a statistical significance level of *p* = 0.05. Because pre- and post-intervention surveys were completed anonymously, within-subject analyses were not conducted.

## 3. Results

**Figure 3:**
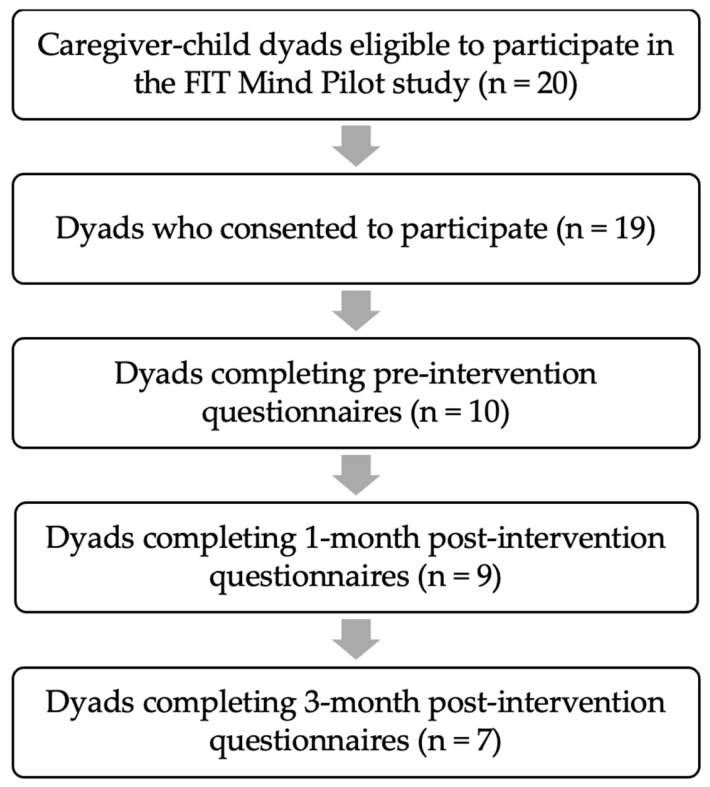
Flow chart of caregiver–child dyads eligible for the Fit Mind pilot study.

Twenty families were eligible to participate in the Fit Mind pilot study from January 2023 through July 2023, and nineteen families consented to participate. The one family that declined to participate in the study still completed Fit Mind outside of the pilot study. Ten participating youth and their caregivers completed the pre-study surveys within one week of their first Fit Mind sessions, nine completed post-study questionnaires one month after program completion, and seven completed post-study questionnaires 3 months after program completion (Figure 3). All questionnaires returned were from complete caregiver–child dyads, and due to this, the number of caregiver and child questionnaires reported were the same at all time points. Participant demographics were not collected as part of this pilot study due to participant privacy concerns.

### 3.1. Fit Mind Feasibility and Feedback

Seventeen of the nineteen (89%) families participating in the study attended all four Fit Mind sessions. Caregivers reported that the Fit Mind program was feasible to participate in due to its telehealth-based format and not having to travel to the clinic to participate. Participants and the Fit Mind facilitator perceived the 30 min length of Fit Mind sessions as an appropriate amount of time to maintain participant attention and to deliver the program’s content. Younger participants (<10 years old) tended to complete the majority of their Fit Mind sessions with a caregiver also participating.

Participants reported positive communication about food choices, health and wellness within their families after completing Fit Mind. Participants specifically described improved decisions regarding food choices (e.g., more home cooked meals, more vegetables consumed), the development of mindful eating practices in the home, and the utilization of mindful relaxation strategies (practicing breathing exercises at home during stressful moments, doing yoga and exercise videos with family members, etc.). During Fit Mind sessions, the Fit Mind instructor noted high levels of participant (youth and caregiver) engagement during most Fit Mind modules. The facilitator perceived participant engagement as being highest during the *Mindful Eating* and *Managing Cravings* sessions, and observed the overall lowest engagement during the third module, *Mindful Thinking*. Most participants were well-engaged during in-session activities (breathing exercises, question-and-answer activities, the mindful eating raisin activity, etc.).

A small percentage of participants had limited engagement in the program (signing into telehealth visits late, multi-tasking during Fit Mind sessions, being out-of-view of the camera during sessions, etc.). Active participation amongst the child participants appeared similar across all ages; some younger children appeared to have greater challenges with distractibility and sitting still during the sessions, and some older teenage participants appeared to have low interest in participating during Fit Mind sessions. Commonly, children were well-engaged with the modules when attending caregivers were also engaged. There was a spectrum of engagement with and reported adherence to the homework assignments, including the weekly family mindfulness-based bingo games, and follow-through on health goals created during Fit Mind sessions. Quantitative data regarding homework completion rates was not collected, due to the high risk of social desirability bias, though in general, many participants reported enjoyment of the bingo homework activities, with multiple family members participating.

Through informal observation, the Fit Mind facilitator observed continued participant engagement when returning for their in-person, multidisciplinary Fit Clinic visits compared to Fit Clinic families who did not receive the Fit Mind programing. Pediatric caregivers of Fit Mind participants generally exhibited higher levels of understanding, engagement, and follow-through with the health and wellness goals generated in clinic. During sessions with the Fit Clinic’s dietitian, Fit Mind pediatric participants reported increased mindful practices while eating (e.g., eating more slowly and recognizing experiencing a food craving when they were not truly hungry and deferring food consumption in those moments). Some caregivers and youth reported at full-team follow-up visits that the whole family began implementing mindful eating strategies at home, such as eating dinner together at the table, eating without devices, and having food-related conversations at home after Fit Mind.

### 3.2. Pediatric Questionnaire Results

Pediatric participants reported an average baseline MEQ-C summary score of 2.4 (SD = 0.6). The average MEQ-C summary score trended upwards 1 month after completing Fit Mind (*p* = 0.46), but did not reach statistical significance. There was no statistically significant change in the MEQ-C summary score 3 months after completing the program from baseline (*p* = 0.50). Regarding the MEQ-C subscales, there were no significant changes in the MEQ-C Awareness or Mindfulness subscale scores at 1 month or 3 months after completing Fit Mind compared to baseline. On the CAMM, pediatric participants had an average score of 22.5 (SD = 7.1) prior to starting Fit Mind. There were no significant changes in CAMM scores at 1 month or 3 months after completing Fit Mind from baseline. Pediatric questionnaire results are summarized in Table 1.

### 3.3. Caregiver Questionnaire Results

Caregivers’ MEQ summary scores averaged 2.8 (SD = 0.2) at baseline. There were no significant changes in MEQ summary scores 1 month (*p* = 0.19) or 3 months (*p* = 0.13) after completing the Fit Mind program compared to baseline. Scores on the MEQ Awareness (*p* = 0.3), Emotional (*p* = 0.22), and Disinhibit (*p* = 0.12) subscales 1 month after completing the program trended towards improvement, though results did not reach statistical significance. Caregiver CAMS-R scores trended upwards 1 month (*p* = 0.47) and 3 months (*p* = 0.40) after program completion compared to baseline, although this did not reach statistical significance. Caregiver questionnaire results are summarized in Table 2.

## 4. Discussion

The preliminary results of this pilot study provide information regarding the feasibility and acceptability of the Fit Mind brief MBI program and highlights family interest in learning about mindfulness during the treatment of pediatric overweight and obesity. MBIs are unique and exciting vehicles in the multimodal treatment of pediatric obesity. Given the interrelatedness of toxic stress and obesity [17], the importance of mindfulness education for children and families seeking treatment for pediatric overweight and obesity cannot be overstated. The Fit Mind program was developed considering psychosocial contributors to the pediatric obesity epidemic, including ACEs and toxic stress. Its focus on mindfulness skill-building aimed to address these underlying risk factors for pediatric obesity. The results of this pilot study help inform future iterations of the Fit Mind program for this clinic’s population, as well as future directions of study.

Overall, Fit Mind was feasible to implement. The Fit Mind program, consisting of two hours of content delivered over four weeks, is significantly shorter than other published MBIs for pediatric weight management [34,35]. Families reported appreciation of the telehealth-based format of content delivery, which may increase the accessibility of this program to families by overcoming transportation and large geographical distance barriers [49]. Given the high attendance rate of all four of the Fit Mind telehealth-based video sessions (89%), technological barriers appeared low in our study population. The overall high session attendance rate highlights the overall acceptability and accessibility of the program among participants.

Over the course of four weeks, it was feasible for clinic staff to review, offer, and discuss specific and practical strategies for bringing awareness to sensations and behaviors around eating and food. The strategies discussed during Fit Mind were family-focused, allowing many opportunities to practice a health goal within the family unit. While no significant differences in validated mindfulness or mindful eating rating scale scores for parent or child participants were observed, it is possible that the brevity of the intervention, while increasing feasibility, may have contributed to the lack of overall change in questionnaire scores post-intervention.

Participant engagement was observed throughout the four modules and appeared to be highest during the *Mindful Eating* and *Managing Cravings* modules. We believe that these modules were best received as they included practical strategies taught using engaging, hands-on activities, such as building awareness around the smell, texture, and taste of food during mealtimes and asking families to complete a corresponding homework assignment to bring together family members to do the same in the home setting (e.g., family Mindful Eating Bingo) through the *Mindful Eating* module. Comparatively, participants overall appeared least engaged with the *Mindful Thinking* module; we suspect this was due to the *Mindful Thinking* module containing the highest-level content for participants to comprehend, based on concepts of the Cognitive Behavioral Triad [50], and included in-session activities, such as ABCD Mindful Thinking, that may be somewhat abstract for some participants. These observations will help guide future modifications to the Fit Mind curriculum, such as identifying more practical and engaging strategies to meet the objectives of *Mindful Thinking*, so that the content is optimized for participant engagement. Beyond the duration of the program, the Fit Clinic team also observed participants continuing to use the mindfulness strategies at subsequent Fit Clinic visits (after the completion of the Fit Mind program). The informal feedback and reflective observations are important considerations for future iterations of this program and a possible area of future study.

### Limitations and Future Research

Several study limitations are recognized in this small, preliminary pilot study. While it was recognized that study recruitment by a member of the clinic team could have influenced families to participate, the team aimed to reduce potential bias by offering families the opportunity to receive the four Fit Mind sessions without participating in the pilot study. The small sample size of this study, from a single clinical site in a large metropolitan area, limits the generalizability of the findings for pediatric patients and families seeking treatment for pediatric obesity. To protect participant privacy, participant demographics were not collected as part of this preliminary pilot study, which further limits the generalizability of our findings. While the absence of demographic data for our study participants is a limitation, the general descriptives of our patient population obtained through prior assessment were included to contextualize the general Fit Clinic patient population.

It should be noted that the reported observations of participant engagement during Fit Mind sessions and subsequent Fit Clinic visits relied on informal observations by team members, which were susceptible to observer bias. Furthermore, the lack of a study control group hinders our ability to determine whether any observed improvements in self-reported healthy eating behaviors or engagement with the Fit Clinic over time were due to the skills learned in Fit Mind or related to the usual care within the Fit Clinic. Further study of this intervention would benefit from a randomized controlled trial to assess causation in evaluating program effectiveness. Another limitation was the lack of fidelity measures in the delivery of the four modules. However, variability in delivery content was minimized since the four modules were delivered in a standardized manner by one facilitator (the Fit Clinic’s licensed clinical social worker) to all participants.

Regarding the study’s exploratory outcomes, the absence of statistically significant changes in total or subscale mindful eating and general mindfulness rating scale scores for child and caregiver participants was likely impacted by the small sample size, with low pre-intervention questionnaire completion, and significantly fewer participants completing questionnaires post-intervention. Both the small sample size and high dropout rate for questionnaire completion significantly increased the risk of Type II errors in analyzing this data. In future study, questionnaire completion rates could be strengthened by having participants complete questionnaires during their first and final Fit Mind sessions, and at follow-up Fit Clinic visits after Fit Mind program completion, rather than independently. Of note, some caregivers did not fully participate in Fit Mind sessions, which may have negatively impacted any change in caregiver questionnaire scores pre- and post-intervention. Additionally, given that questionnaires were completed anonymously to protect participant privacy, the inability to conduct within-subject analyses further limits interpretation of pre- and post-intervention questionnaire results.

The quantitative exploratory outcomes from participant questionnaires relied on self-report, introducing a risk of response bias. Although the questionnaires used in this pilot study have previously published validity evidence [43,44,46,47,48], not all questions on the MEQ-C, MEQ, CAMM, and CAMS-R questionnaires were directly related to the specific content presented during Fit Mind. Despite this limitation, the four questionnaires used in this study were chosen for their published evidence of acceptable psychometrics and their ease of distribution and completion by families, with feasibility for use in a larger future study. Questionnaires additionally did not take the specific individual or family-level behavioral changes targeted through the Fit Mind intervention into consideration; these would be interesting to investigate in future studies, along with objective outcomes related to pediatric obesity management, such as anthropometrics and lab markers, in clinical control trial evaluations of pediatric obesity interventions.

## 5. Conclusions

It is important to consider the impact of, and provide treatment to combat, toxic stress in providing comprehensive management of pediatric obesity. MBIs have been identified as a promising intervention to support patients being treated for obesity. The primary goal of this pilot study was to assess the acceptability and feasibility of a brief, evidence-informed mindfulness curriculum in the treatment of pediatric obesity, in a busy, multi-disciplinary weight management clinic. Participants demonstrated interest and engagement in program content based on facilitator observation and high program attendance, thus supporting the feasibility, acceptability and accessibility of such an intervention. The lessons learned through this preliminary pilot study provide valuable insights for the next iteration of the Fit Mind program and future MBI programs under development. More research is needed to evaluate the impact of the Fit Mind mindfulness program on pediatric patients seeking treatment for overweight and obesity, including comparing anthropometrics, vital signs and biomarker levels (Figure 1), along with general mindfulness and mindful eating outcomes, pre- and post-intervention, in a larger, controlled trial. This future work will help guide the medical community in providing the most effective care to support this patient population.

## Figures and Tables

**Table 1:** Comparison of Pediatric Participant Mean (SD) Questionnaire Results at Baseline to One Month and Three Months Post-Intervention.

MEQ-C	Baseline M (SD); *n* = 10	1-Month M (SD); *n* = 9	*p*-Value ^a^	3-Months M (SD); *n* = 7	*p*-Value ^b^
MEQ-C Summary Score	2.4 (0.6)	2.5 (0.6)	0.46	2.4 (0.7)	0.50
Awareness Subscale	2.9 (0.4)	2.9 (0.7)	0.48	3.0 (0.9)	0.42
Mindfulness Subscale	2.2 (0.8)	2.3 (0.9)	0.45	2.2 (0.8)	0.47
**CAMM**	**Baseline** **M (SD); *n* = 10**	**1-Month** **M (SD); *n* = 9**	***p*** **-Value ^a^**	**3-Months** **M (SD); *n* = 7**	***p*** **-Value ^b^**
CAMM Score	22.5 (7.1)	22.0 (10.0)	0.45	19.3 (9.2)	0.21

^a^ *p*-value comparing scores at baseline to 1 month post-intervention; ^b^ *p*-value comparing scores at baseline to 3 months post-intervention. MEQ-C = Mindful Eating Questionnaire—Child; CAMM = Child and Adolescent Mindfulness Measure.

**Table 2:** Comparison of Caregiver Participant Mean (SD) Questionnaire Results at Baseline to One Month and Three Months Post-Intervention.

MEQ	Baseline M (SD); *n* = 10	1-Month M (SD); * n* = 9	*p*-Value ^a^	3-Months M (SD); *n* = 7	*p*-Value ^b^
MEQ Summary Score	2.8 (0.2)	2.8 (0.2)	0.19	2.6 (0.2)	0.13
Awareness Subscale	2.7 (0.4)	2.8 (0.5)	0.30	2.4 (0.4)	0.13
Distract Subscale	2.7 (0.4)	2.7 (0.6)	0.45	2.5 (0.7)	0.21
Emotional Response Subscale	2.9 (0.7)	3.1 (0.8)	0.22	2.9 (0.5)	0.40
External Cues Subscale	2.6 (0.3)	2.5 (0.6)	0.35	2.4 (0.4)	0.13
Disinhibition Subscale	2.8 (0.4)	3.0 (0.5)	0.12	2.7 (0.3)	0.35
**CAMS-R**	**Baseline** **M (SD); *n* = 10**	**1-Month** **M (SD); *n* = 9**	***p*** **-Value ^a^**	**3-Months** **M (SD); *n* = 7**	***p*** **-Value ^b^**
CAMS-R Score	30.2 (5.7)	30.4 (8.2)	0.47	30.9 (4.9)	0.40

^a^ *p*-value comparing scores at baseline to 1 month post-intervention; ^b^ *p*-value comparing scores at baseline to 3 months post-intervention. MEQ = Mindful Eating Questionnaire; CAMS-R = Cognitive and Affective Mindfulness Scale—Revised.

## Data Availability

The datasets used and analyzed in this study are available from the corresponding author upon reasonable request.

## Abbreviations

The following abbreviations are used in this manuscript:

BMI	Body mass index
ACE	Adverse childhood experiences
PEARLS	Pediatric ACEs and Related Life Events Screener
MBI	Mindfulness-based intervention
MEQ-C	Mindful Eating Questionnaire-Child
CAMM	Child and Adolescent Mindfulness Measure
MEQ	Mindful Eating Questionnaire
CAMS-R	Cognitive and Affective Mindfulness Scale-Revised
